# A New Solution to a Rare Problem of Implantable Cardiac Device Hypersensitivity

**DOI:** 10.7759/cureus.17882

**Published:** 2021-09-10

**Authors:** Rana Al-Zakhari, Safa Aljammali, Ryan Isber, Angela Grigos, Nidal Isber

**Affiliations:** 1 Internal Medicine, Richmond University Medical Center, New York, USA; 2 Biology, Bringhamton University, New York, USA; 3 Internal Medicine/Cardiology, Richmond University Medical Center, New York, USA

**Keywords:** implantable cardiac defibrillator (icd), cardiovascular implantable electronic device (cied), pacemakers (pm), acid fast bacilli (afb), antibiotic coated envelope

## Abstract

For this research, we have considered a case of a man aged 60 years who developed painless fluid accumulation in the implantable cardiac defibrillator (ICD) pocket site. The cardiovascular implantable electronic device initially appeared to be infected, but it was eventually determined that the cause was an allergic reaction, and a novel solution was implemented. For patients with nickel allergies, treatment typically includes avoiding nickel or replacing with gold-plated devices with new leads. Because of the subclavian vein thrombosis, the gold-plated generator was not replaced. ICD generators were covered with antibiotic-coated envelopes while waiting for the replacement. Hypersensitivity to cardiac devices was effectively treated with this technique. By routinely using the envelope, the very rare incidence of device hypersensitivity could further be reduced.

## Introduction

It was reported from 1993 to 2008 that there were 4.2 million primary pacemaker implantations (3,204,700 records) and 1,124,000 primary implantable cardiac defibrillator (ICD) implantations (1,124,200 records), and a cardiovascular implantable electronic device (CIED) can cause an allergic reaction, though it is uncommon [1,2]. We report a case of an allergy to a component of the CIED, with recurrent fluid accumulation in the device pocket that was successfully treated after coating the device with an antibiotic envelope.

## Case presentation

**Figure 1:**
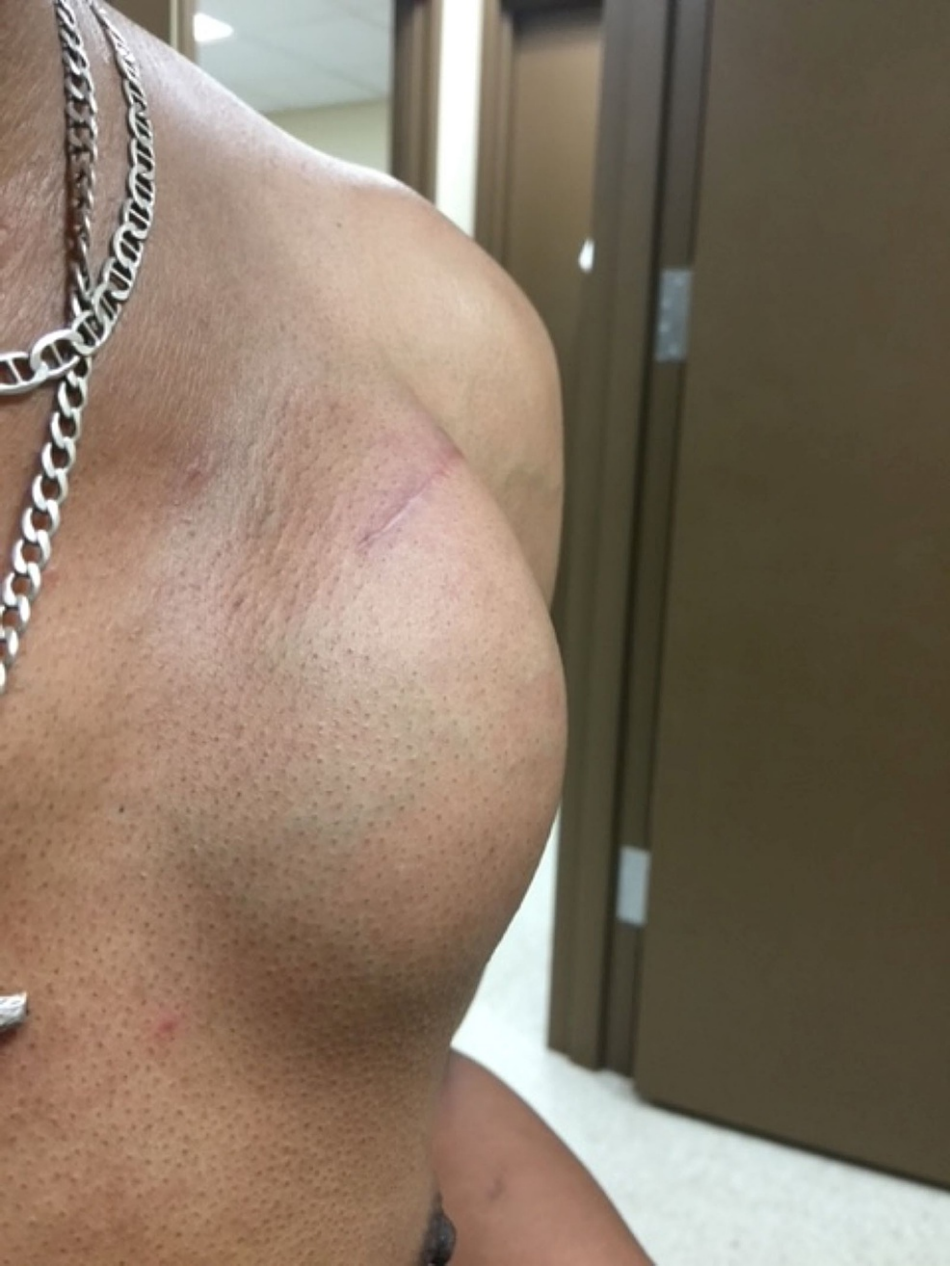
ICD pocket site swelling ICD, implantable cardiac defibrillator

**Figure 2:**
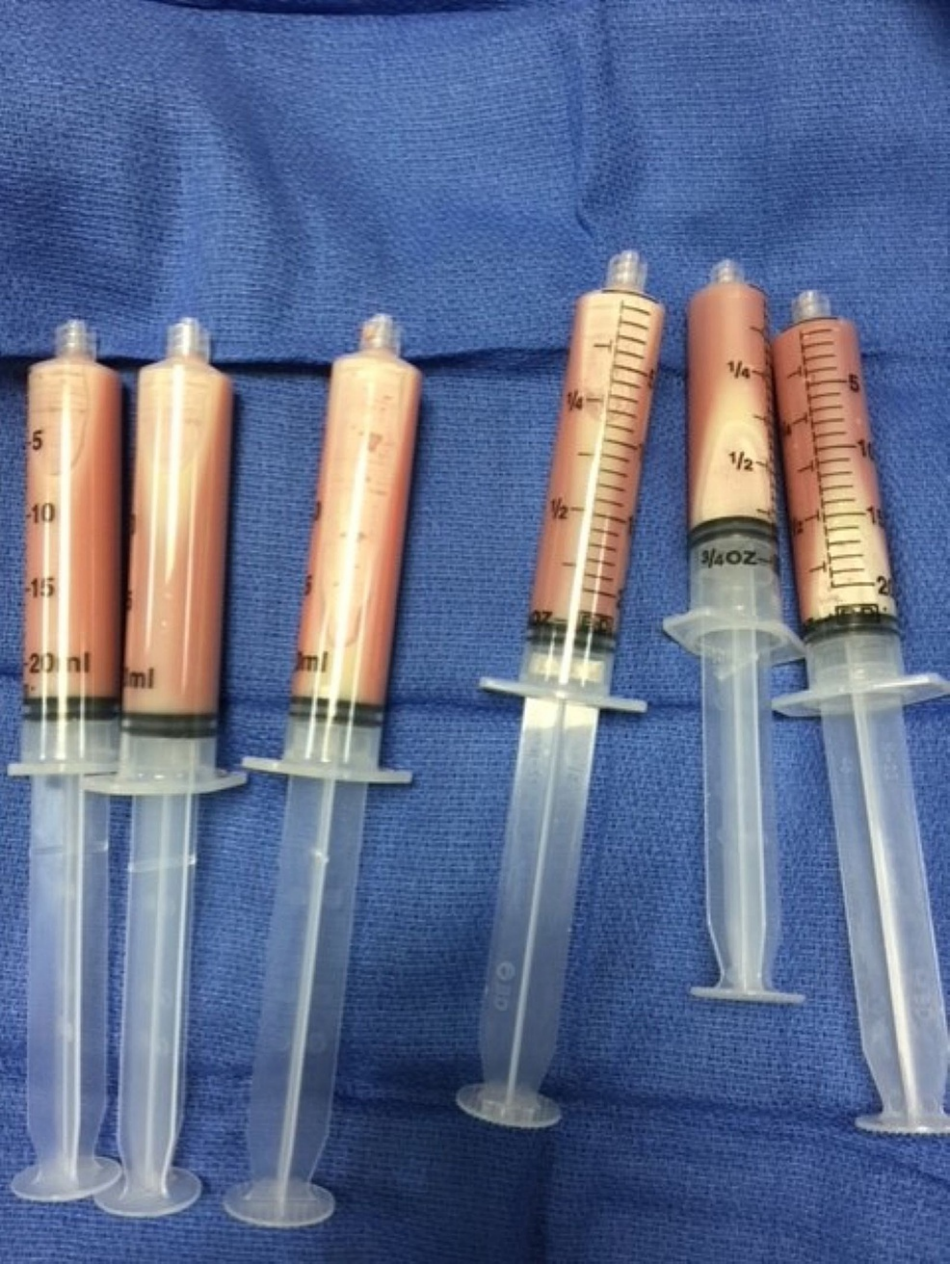
ICD pocket fluid aspirate ICD, implantable cardiac defibrillator

The patient is a 60-year-old African American male who survived cardiac arrest due to ventricular tachycardia which necessitated the need for a dual-chamber ICD. Placement was without any complications. However, three weeks later, the site of the device developed swelling, with tense fluctuating fluid accumulation (Figure 1). No other local signs of infection were present, such as erythema, tenderness, or increased warmth of the skin. There were neither systemic signs nor any symptoms of infection, such as fever, tachycardia, or leukocytosis. Over the course of the next three months, the pocket was aspirated twice, drawing 140 cc of fluid each time (Figure 2). However, within 12 to 24 hours of each aspiration the fluid re-accumulated to the same degree. Pocket fluid analysis revealed white blood cell count of 1215/mL (88% was lymphocytes) (Table 1). Numerous blood and fluid cultures, gram stains, and acid-fast bacilli statins returned negative (Table 2). The lack of infective organism and the rapidity of fluid accumulation strongly suggested a diagnosis of ICD hypersensitivity. Skin patch test revealed sensitivity to nickel. The only reliable treatment was the removal of the system component. It was then decided to remove the ICD system and to implant a gold-plated generator. During the procedure, inspection of the pocket after removal of the fluid revealed no signs of infection. The attempt to implant a new lead that was compatible with the gold-plated generator was not possible due to subclavian vein thrombosis. Due to worries of complications, such as pneumothorax, the procedure was aborted, and right-side device implantation was planned for a later date. Meanwhile, during this attempt, an antibiotic-coated envelope was used to entirely coat the ICD generator, to prevent device-tissue interaction. The antibiotic envelope consisted of the antimicrobials minocycline and rifampin and therapeutic levels eluted into the ICD pocket site. Follow-up at one week, four weeks, and three months showed no re-accumulation of fluid. The patient continued to be completely asymptomatic and the patient did not receive any systemic antibiotics.

**Table 1:** ICD pocket aspirate fluid analysis findings WBC, white blood cell count; LDH, lactate dehydrogenase; ICD, implantable cardiac defibrillator

ICD Pocket Fluid Aspirate Parameters	Results
Fluid WBC	1215/μL, 88% lymphocytes
Fluid glucose	102 mg/dL
Total protein	3.6 g/dL
Fluid albumin	2.4 g/dL
Fluid LDH	124 U/L
Fluid amylase	44 U/L

**Table 2:** Microbiology results from ICD pocket fluid aspirate ICD, implantable cardiac defibrillator

Microbiology Parameters	Results
Gram stain	No organisms seen, white blood cells few
Culture	No acid-fast bacilli seen, no growth after six weeks

This case demonstrates that fluid accumulation, unresolved after repeated aspiration, could be indicative of hypersensitivity reaction to a component of the ICD such as nickel, as in this case. Entirely coating the device with an antibacterial envelope is an effective way to treat this complication.

## Discussion

CIEDs provide a lifesaving therapy for the treatment of bradyarrhythmias, ventricular tachyarrhythmias, and advanced systolic heart failure. These devices are made up of of three major components: device pulse generator, device controller-monitor, and leads. These components are usually coated with various materials such as silastic, titanium, nickel, polyurethane, epoxy, mercury, cadmium, chromate, silicone, polychloroparaxylene, and cobalt to reduce infections and reaction [2].

Allergies to cardiac devices are relatively uncommon conditions that result from contact hypersensitization to the coating material of the device components. Among a few, the incidence rate was about 571 per 1 million in 1997 [2,3]. 

The underlying mechanism of this complication is still not well understood, but most likely related to genetics of human leucocyte antigen, as the sensitivity reaction can occur to multiple materials. There can be a delay between the time of implant and the time of onset of symptoms, which include edema, erythema, bullae, vesicles, and plaque, the most apparent manifestations of the allergic reaction [2,3]. These patients may develop hypotension and fever similar to those associated with CIED infection. An allergy to a device should only be considered after a thorough examination of the patient and any infectious process has been ruled out [4].

Although there are no definite guidelines for treating CIED hypersensitivity reaction, replacing the CIED device with non-allergic components like gold-plated generator has been a usual treatment option. But, removing the device is still the only definitive method of treatment [5,6]. A novel approach to treating this condition is presented in our case. Herein, we used antibiotic envelope to completely cover the generator to avoid contact between the device and tissue.

## Conclusions

Our case introduces an alternative way to treat this complication. Our use of the antibiotic envelope was intended to completely coat the whole generator to prevent device-tissue interaction. However, we do not routinely use this in our lab due to our extremely low infection rate. The widespread routine use of the envelope might further decrease the already very rare incidence of device hypersensitivity. This was a successful option for treatment in the case of our patient.
